# Supplementary material for: Multifunctional Gelatin-Nanoparticle-Modified Chip for Enhanced Capture and Non-Destructive Release of Circulating Tumor Cells
Source: Micromachines (Basel). 2022 Feb 28;13(3):395. doi: 10.3390/mi13030395 (PMC8955365; doi:10.3390/mi13030395)
Supplement: Supplementary file 1 [file micromachines-13-00395-s001.zip › micromachines-1572331-supplementary.pdf]

Supplementary

Multifunctional Gelatin-Nanoparticle-Modified Chip for Enhanced Capture and Non-Destructive Release of Circulating Tumor Cells

Linying Xu [1], Tiantian Ma [2], Kelin Zhang [1], Qilin Zhang [1], Mingxia Yu [2,*] and Xingzhong Zhao [1,*]

[1] Key Laboratory of Artificial Micro- and Nano-structures of Ministry of Education, School of Physics and Technology, Wuhan University, Wuhan 430072, China; xulinying@whu.edu.cn (L.X.); 9176605@163.com (K.Z.); qilin-zhang@whu.edu.com (Q.Z.)

[2] Department of Clinical Laboratory, Zhongnan Hospital of Wuhan University, Wuhan 430071, People's
Republic of China; 2020203030021@whu.edu.com

* Correspondence: xzzhao@whu.edu.cn (X.Z.); dewrosy520@whu.edu.cn (M.Y.)

1. Characterizations supplementary

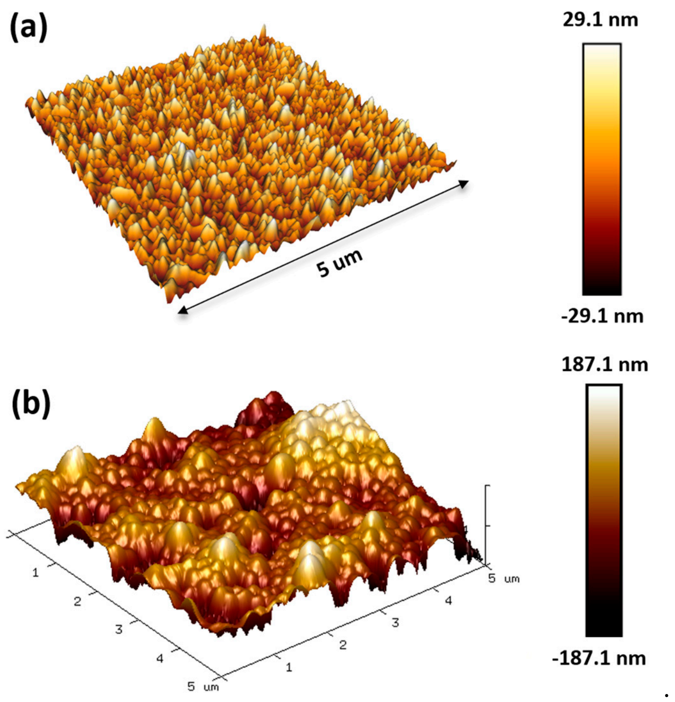

Figure S1. (a)AFM image for bare FTO substrate; (b) AFM image for GNPs coated substrate.

2. Cell capture supplementary

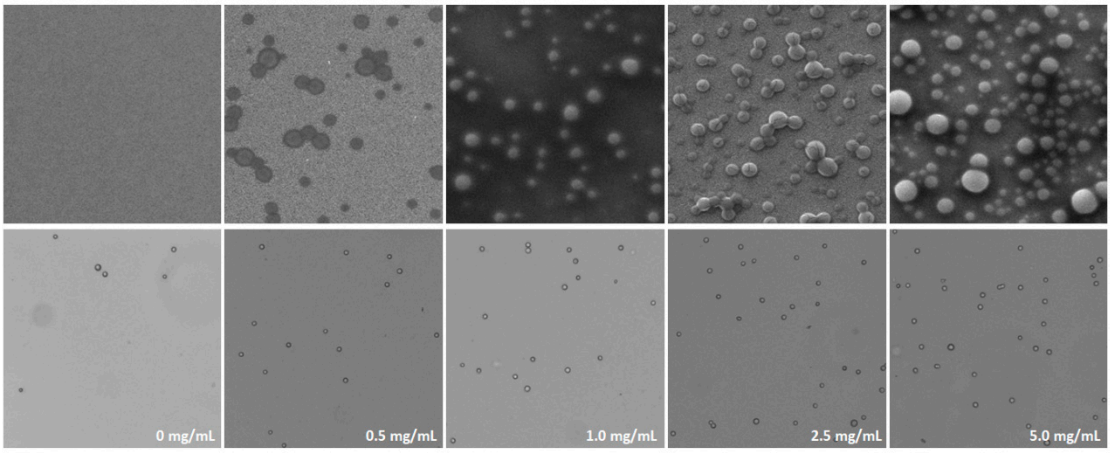

2.

Figure S2. Characterization of 5 different concentrations of gelatin nanoparticles (0, 0.5, 1.0, 2.5, 5.0 mg/mL) modified chips and performance on MCF-7 cells capture.

Table S1. Capture efficiency of different concentrations of GNPs modified chip for MCF-7.

Concentrations of GNPs	0 mg/mL	0.5 mg/mL	1.0 mg/mL	2.5 mg/mL	5.0 mg/mL
Capture efficiency	25.17%±7.68%	50.60%±3.25%	74.71%±4.22%	89.27%±0.38%	89.57%±2.20%

Table S2. Cell capture experiment details with MCF-7, HCT116 and WBCs to evaluate the specificity of anti-EpCAM

Type of cell	Spiked number (in PBS)	Captured number	Capture efficiency	Average capture efficiency
MCF-7	1000	874	87.40%	89.57%
	1000	918	91.80%	
	1000	895	89.50%	
HCT116	1600	1343	83.94%	88.17%
	1600	1427	89.19%	
	1600	1462	91.38%	
WBCs	169200	9053	5.35%	5.75%
	169200	10890	6.44%	
	169200	9269	5.48%	

Table S3. Specific series number of MCF-7 cells spiked in PBS and mononuclear cells to vadiate the capture performance.

MCF-7 cells spiked in PBS		MCF-7 cells spiked in processed blood	
Number of spiked cells	Number of capture cells	Number of spiked cells	Number of capture cells
15	13	25	18
32	28	28	25
68	42	65	63
118	103	113	100
178	136	161	140

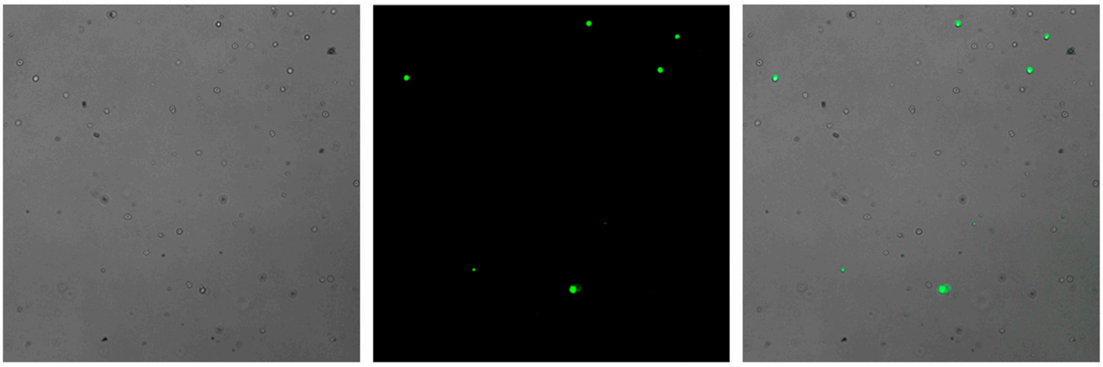

Figure S3. Fluorescent images of the captured MCF-7 cancer cell lines spiked in processed blood.

3. Cell release supplementary of MCF-7 cell lines and peripheral blood mononuclear cells (PBMCs)

Table S4. Cell viability test of released MCF-7 cell lines. the numbers of live cells and dead cells are shown under conditions of different MMP-9 concentrations.

MMP-9 concentration	Viable cells	Dead cells	Viability	Average
0 mg/mL (control group)	463	6	98.72%	98.29%
	565	9	98.43%	
	300	7	97.72%	
0.01 mg/mL	565	9	98.43%	98.00%
	300	7	97.72%	
	500	11	97.85%	
0.1 mg/mL	620	10	98.41%	98.38%
	418	7	98.35%	
	488	8	98.39%	
0.2 mg/mL	74	2	97.37%	96.91%
	89	2	97.80%	
	86	4	95.56%	
0.5 mg/mL	463	6	98.72%	98.09%
	435	12	97.32%	
	554	10	98.23%	

Table S5. Cell viability of released peripheral blood mononuclear cells (including CTCs) from a gastric cancer patient (female 69).

Microscope field No.	Dead cells	Alive cells	Cell viability
1	7	47	
2	16	42	
3	12	35	
4	9	36	
5	17	30	
6	34	65	
7	15	37	
8	11	60	
9	6	28	
10	10	35	
11	11	22	
12	5	33	

13	8	20	
14	5	25	
15	2	11	
16	7	26	
17	5	45	
18	15	83	
19	14	79	
20	25	92	
total	234	851	78.43%

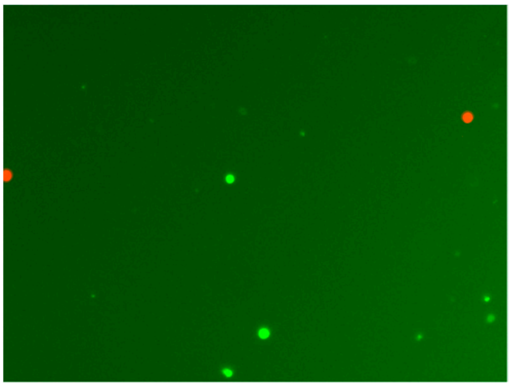

Figure S4. Part of a microscope field to show details about released peripheral blood mononuclear cells (including CTCs). The green fluorescent cells are FDA-stained live cells, and red are PI-stained dead cells.

4. Information for Cancer Patients and Healthy individuals

Table S6. The information of cancer patients and healthy individuals.

Patient number	Cancer type	Age	Gender	CTC numbers (1 mL blood)
#1	breast cancer	50	female	13
#2	breast cancer	48	female	11
#3	breast cancer	51	female	4
#4	colorectal cancer	57	female	7
#5	colorectal cancer	55	male	6
#6	colorectal cancer	65	male	10
#7	colorectal cancer	59	male	3
#8	Healthy individuals	24	female	0
#9	Healthy individuals	65	male	0
